# Fabrication of AO/LDH fluorescence composite and its detection of Hg^2+^ in water

**DOI:** 10.1038/s41598-017-13779-1

**Published:** 2017-10-17

**Authors:** Meng Liu, Guocheng Lv, Lefu Mei, Yanke Wei, Jieyuan Liu, Zhaohui Li, Libing Liao

**Affiliations:** 1 Beijing Key Laboratory of Materials Utilization of Nonmetallic Minerals and Solid Wastes, National Laboratory of Mineral Materials, School of Materials Science and Technology, China University of Geosciences, Beijing, 100083 P. R. China; 2 Geosciences Department, University of Wisconsin – Parkside, Kenosha, WI 53144 USA

## Abstract

Divalent mercury ion (Hg^2+^) is one of the most common pollutants in water with high toxicity and significant bioaccumulation, for which sensitive and selective detection methods are highly necessary to carry out its detection and quantification. Fluorescence detection by organic dyes is a simple and rapid method in pollutant analyses and is limited because of quenching caused by aggregation dye molecules. Hydrotalcite (LDH) is one of the most excellent carrier materials. In this study, an organic dye acridine orange (AO) was successfully loaded on the LDH layers, which significantly inhibited fluorescence quenching of AO. The composite AO/LDH reaches the highest fluorescence intensity when the AO initial concentration is 5 mg/L. With its enhanced fluorescent property, the composite powder was fabricated to fluorescence test papers. The maximal fluorescence intensity was achieved with a pulp to AO/LDH ratio of 1:5 which can be used to detect Hg^2+^ in water by naked eyes. Hg^2+^ in aqueous solution can be detected by instruments in the range of 0.5 to 150 mM. The novelty of this study lies on both the development of a new type of mineral-dye composite material, as well as its practical applications for fast detection.

## Introduction

Mercury is widespread pollutant, which exists as metallic, inorganic, and organic species in environment^1^. Hg^2+^ ion is one of the most prevalent forms of mercury species in environmental waters^2,3^, which can further transform into the more toxic methylmercury by microbial methylation^4,5^. Hg^2+^ ion can accumulate in animal and human bodies through food chains^6^, with a high enrichment factor, and then affect the nerves, immune, and digestive systems and cause serious damage of kidney, liver, and brain^7,8^. Hence, there is a great need to develop a simple and rapid method for detection of trace Hg^2+^ ion in water.

The traditional techniques for Hg^2+^ ion detection include cold vapor atomic absorption spectrometry (CVAAS)^9,10^, inductively coupled plasma atomic emission spectrometry (ICPAES), atomic fluorescence spectrometry (AFS)^11^, liquid chromatography inductively coupled plasma mass spectrometry (LCICPMS)^12^, and anodic stripping voltammetry (ASV)^13,14^. Although these methods are reliable and sufficiently sensitive for routine analysis, there are several shortcomings, such as the need for expensive equipment, well-trained operators and complicated sample preparation, and not being competent for on-site detection in emergent pollution events^8,15^. Thus, a simple and rapid method to analyze Hg^2+^ concentrations in water will be of great importance.

Acridine orange (AO) is a nucleic acid selective fluorescent cationic dye which is often used to probe DNA structure in drug-DNA and protein-DNA interactions^16^. However, its application on florescent detection was limited because of quenching^17,18^. Thus, stabilization and enhancement of their luminous intensity are critical when applied in solid phase. Although several methods for the assembly of flexible film materials involving in using small organic molecules and organic polymers have been developed, they usually suffer from phase separation, aggregation of small organic molecules, or decreased fluorescence quantum yield^19^. The combination of different materials is an excellent way to improve their performance^20^. An innovative method to stabilize small organic molecules of luminescence was to intercalate them into the interlayer of saponite clay^21^. Such an inorganic-organic route enhanced the physical stability and compatibility of the dyes and could be used in application of fluorescence detection.

Hydrotalcites (LDH), a family of two-dimensional anionic layered materials, have attracted extensive interests, because of their novel properties and extensive application for catalysts, molecular container, flame retardants, acid absorbents, drug delivery, etc^22–25^. LDH are minerals with a high anion exchange capacity in contrast to the well-known clay minerals^26^, which have cation exchange properties^27^. The structure of these minerals consists of brucite-like positively charged layers resulting from partial substitution of the original Mg^2+^ by Al^3+^ ^28^. The positive charges are compensated by inorganic anions. In the natural form, LDH are hydroxycarbonates of magnesium and aluminum with the formula: [Mg_6_^2+^Al_2_^3+^(OH)_16_]^2+^CO_3_^2−^·4H_2_O^29^.

In this study, we prepared a fluorescence test paper for the detection of Hg^2+^. Due to quenching effect, the photoactive molecule AO was loaded on the surface of LDH to reach maximal separation and minimal aggregation. Fluorescence quenching was assessed using variety of solutions. The AO/LDH fluorescence test paper resulted in a superior response to Hg^2+^ detection.

## Results and Discussion

### Preparation Characterization of AO/LDH

**Figure 1:**
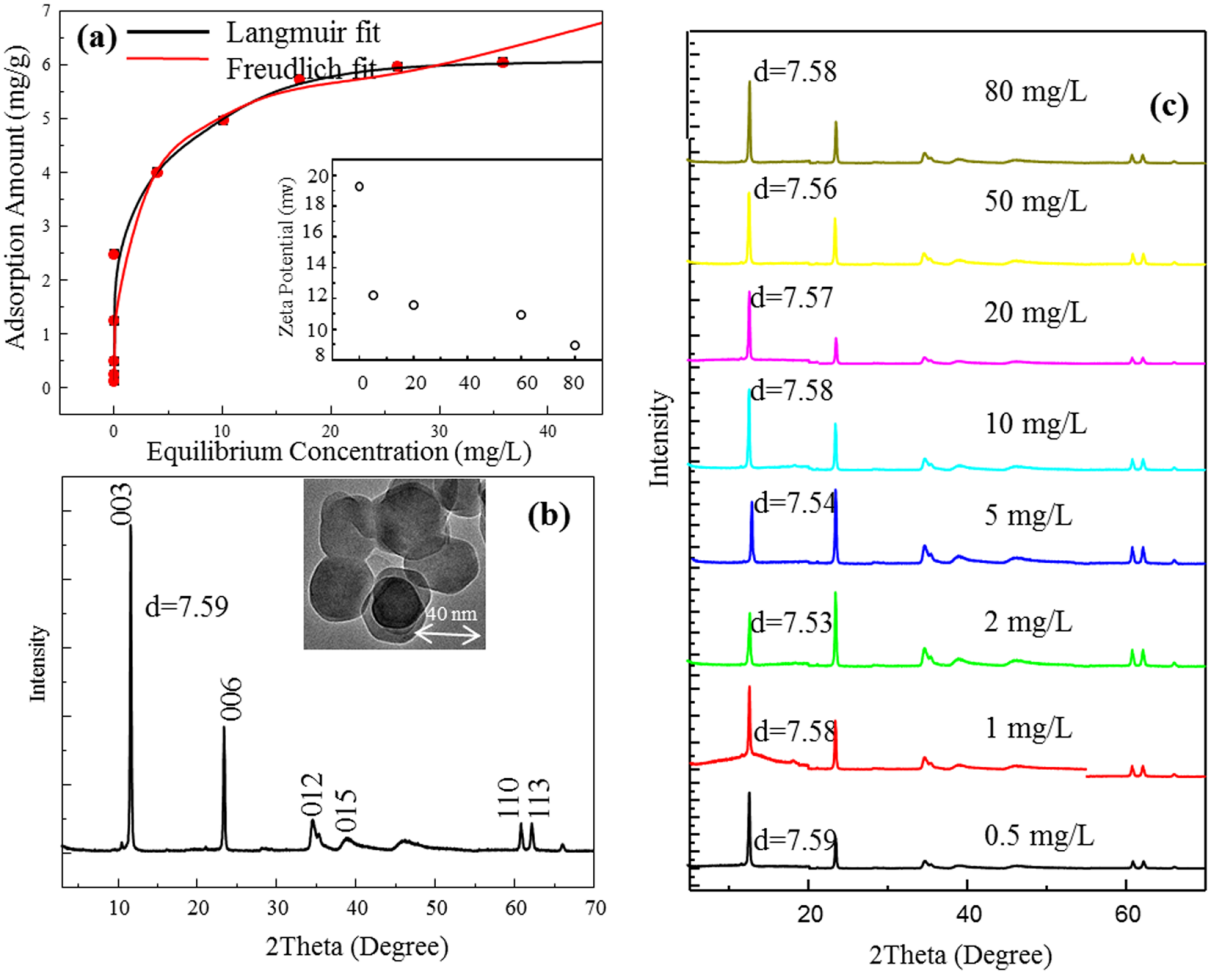
AO adsorption isotherm on LDH (**a**) and Zeta potential of LDH after AO adsorption from different initial concentrations (inset). Characterization of raw LDH by X-ray diffraction (**b**) and TEM (inset). X-ray diffraction patterns of LDH as affected by initial AO concentrations (**c**).

The AO adsorption data were fitted using the Langmuir and Freundlich adsorption models (Fig. 1a). The Langmuir^30^ equation can be described as:

1 $$\frac{1}{{q}_{e}}=\frac{1}{{q}_{max}{K}_{L}{C}_{e}}+\frac{1}{{q}_{max}}$$

where q_e_ (mmol·g^−1^) and q_max_ (mmol·g^−1^) are the amount of AO adsorbed at equilibrium time and the maximum quantity of AO per unit clay to form a complete monolayer on the surface, respectively. C_e_ (mmol·L^−1^) is the equilibrium solute concentration. K_L_ (L·mmol^−1^) is Langmuir affinity, which represents enthalpy of sorption and related to temperature^31^.

The Freundlich^32^ model has the form:

2 $$ln{q}_{e}=\,\mathrm{ln}\,{K}_{F}+\frac{1}{n}ln{C}_{e}$$

where K_F_ and *n* are the Freundlich constants related to the sorption capacity and sorption intensity, respectively^33^. The r^2^ was 0.98 when the data were fitted to the Langmuir model. The AO adsorption capacity was 6 mg/g. In comparison, the Freundlich isotherm model which involves multilayer adsorption on adsorption surface fitted the experimental data poorly, with an r^2^ value of 0.93, and therefore was not adopted in this study. The positive properties of the surface are decreased after adsorption, indicating that some molecules are loaded onto the surface of LDH (Fig. 1a inset). The XRD patterns showed reflections of LDH at 2θ = 12.5°, 22.2°, 34.6°, 38.6°, 46.4°, 60.4°, and 61.6°, corresponding to (003), (006), (012), (015), (018), (110), and (113) planes respectively^34^ (Fig. 1b), which are typical for crystallized Mg-Al LDH (CO_3_^2−^) phase. AO was retained on the surface of LDH instead of intercalated into the interlayer of LDH (Fig. 1c).

**Figure 2:**
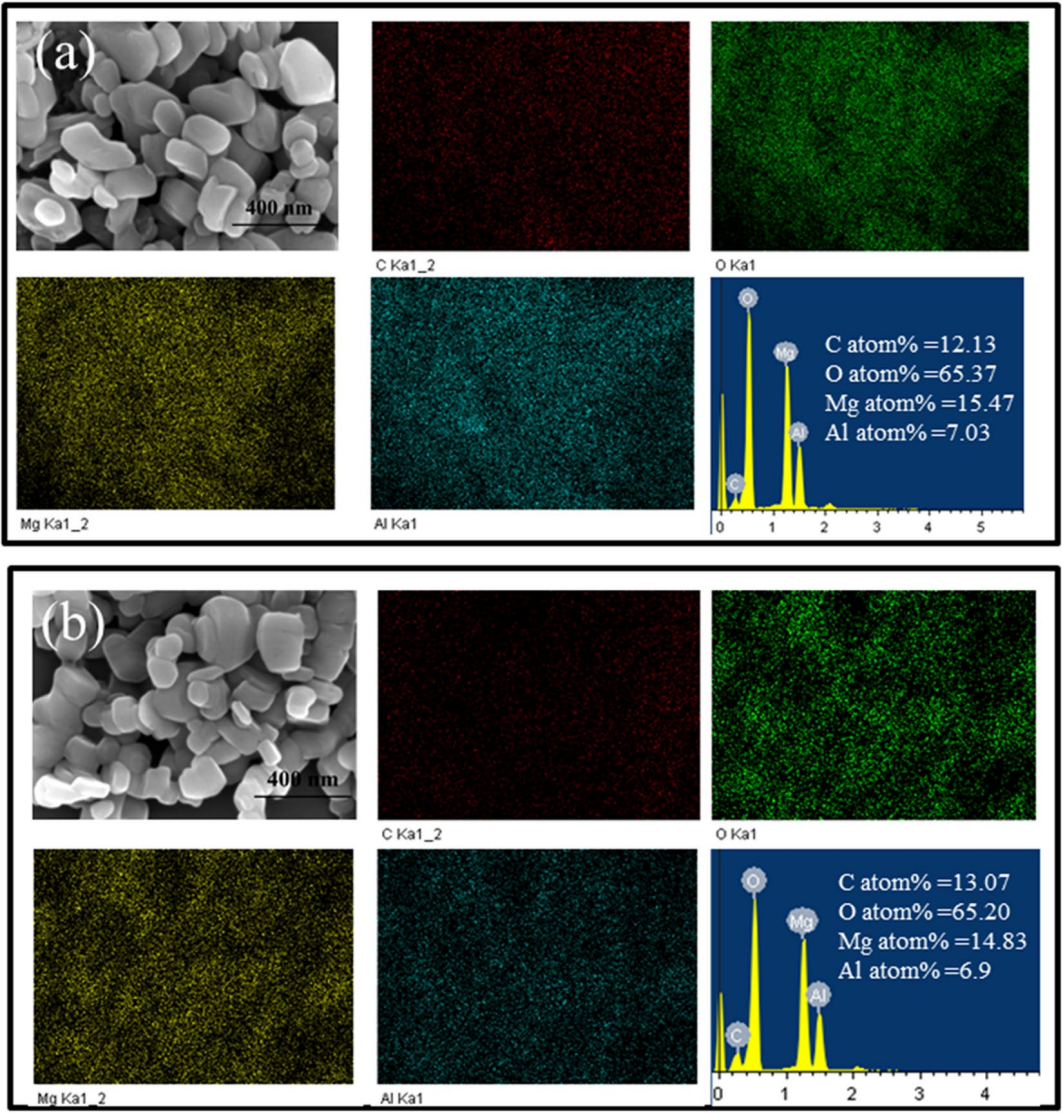
SEM and EDS of the raw LDH (**a**). SEM and EDS of AO/LDH (**b**).

The morphologies of raw LDH and LDH after AO adsorption were characterized by SEM (Fig. 2). The samples are quite similar in shape (tabular) and uniform in size (40–500 nm). No changes in crystal morphology, nor in crystal size was observed after AO uptake on the LDH, suggesting no particle disintegration after upload of AO on LDH (Fig. 2b).

### The luminescence properties of AO/LDH and fluorescence test paper (FTP)

**Figure 3:**
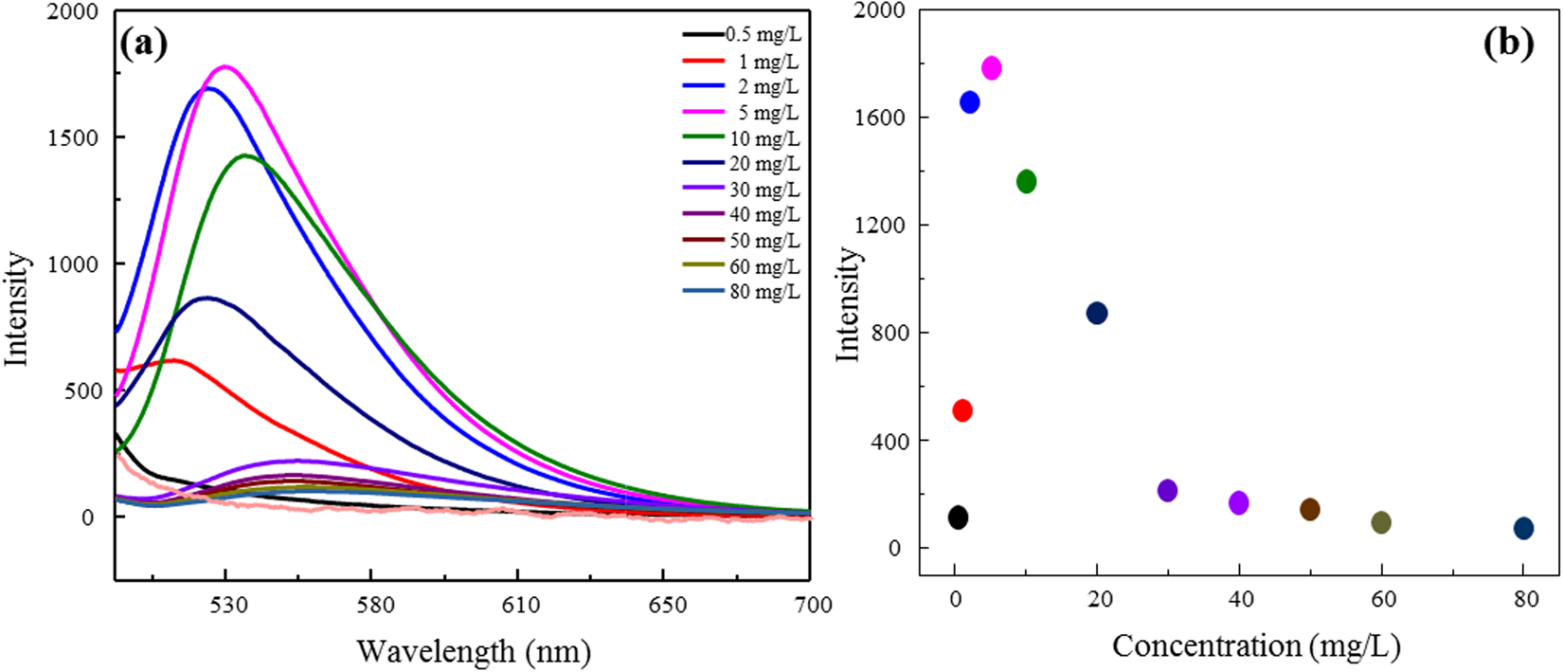
Fluorescence spectra of AO/LDH as affected by the initial AO concentrations (**a**). The peak spectral intensity of AO/LDH under different initial AO concentrations (**b**).

Due to the lack of strong luminescence, AO powder was rarely used as a luminescent material. This very low luminescence intensity was attributed to concentration quenching^35^. After being loaded on the surface of LDH, the luminescence intensity improved significantly (Fig. 3a), perhaps as a result of separation of AO molecules on the surface of LDH. LDH is equal to a solid dispersant which can disperse AO successfully. Thereby the radiationless transition reduces. The raw LDH is not luminous. The fluorescence intensity increases gradually with the increase of AO when different concentrations of AO are dispersed on the layer of LDH. The luminescence intensity of AO/LDH increased as the initial AO concentration increased to 5 mg/L (or at the AO loading of 4 mg/g), at which the highest luminescence intensity of AO/LDH was obtained (Fig. 3b). However, AO fluorescence molecules continue to reunite as the concentration of AO increase resulting in luminescence intensity decreasing.

**Figure 4:**
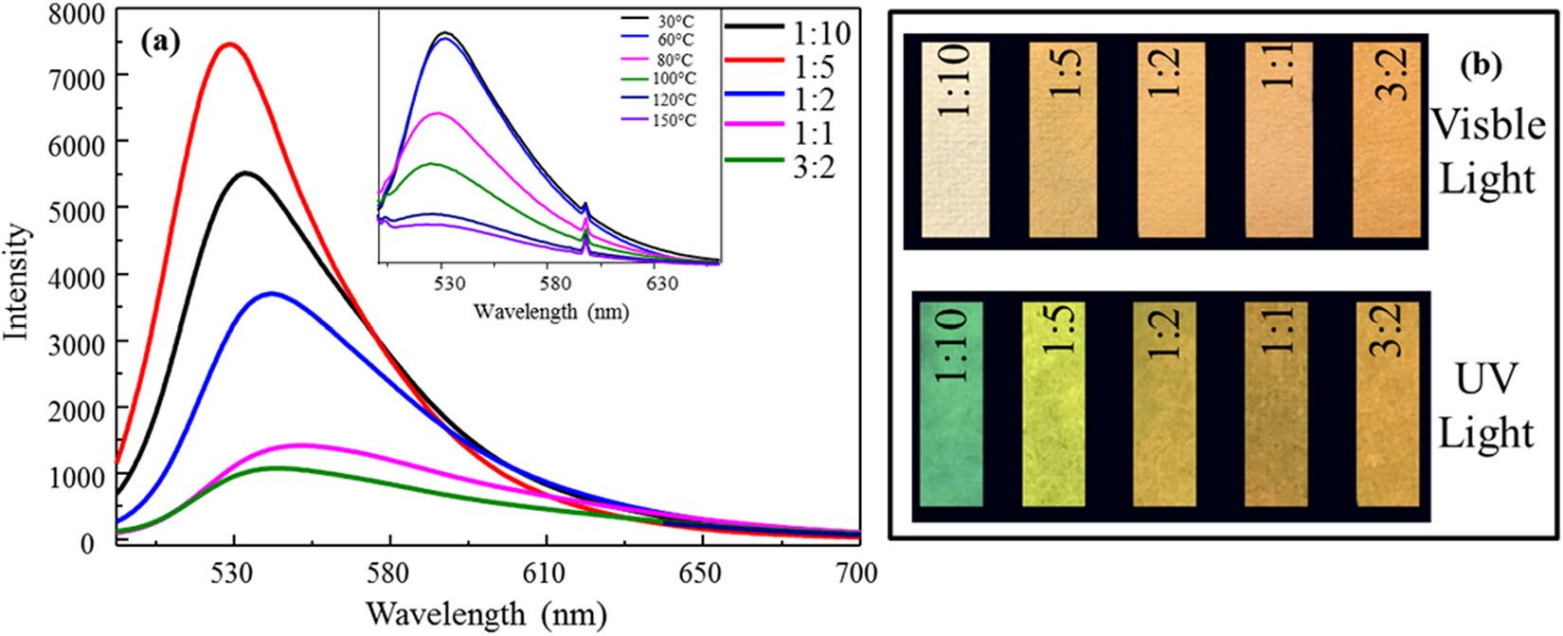
Fluorescence spectra of the FTP made of different ratios of pulp to AO/LDH (**a**) and FTP fluorescence spectra as affected by temperature (inset). Images of FTP under visible and UV lights (**b**).

To fabricate test strips similar to a litmus paper, pulp needs to be added. The amount of pulp added affected the performance of AO/LDH (Fig. 4). Visualization of FTP prepared with different pulp to AO/LDH ratios under visible and UV lights (Fig. 4b) agreed well with the data in Fig. 4a. The AO/LDH power can be further dispersed when combing with pulp. However, it is the best proportion when the ratio of pulp to AO/LDH is 1:5 to achieve the largest luminous intensity (Fig. 4a). Thus, the pulp to AO/LDH ratio of 1:5 was used for fabrication of the FTP to be used to detection of other solutes in aqueous solution. The luminescence property of the composite material changed significantly under different temperatures, suggesting that the thermal stability of the materials is a principal factor affecting its application. The temperature rise reduces the luminescence efficiency of the material, which is called temperature quenching. Quenching means that there is no radiative transition between the electron levels. The rate of radiationless transition increases with temperature, causing the temperature quenching. Despite the luminous intensity of FTP weakened as temperature increased, it did not disappear until about 120 °C (Fig. 4a inset). AO, loaded on the layer of LDH, is under the protection of LDH layer, making it better adaptive to changing ambient temperature.

### Screening and quantification of Hg^2+^ by FTP

**Figure 5:**
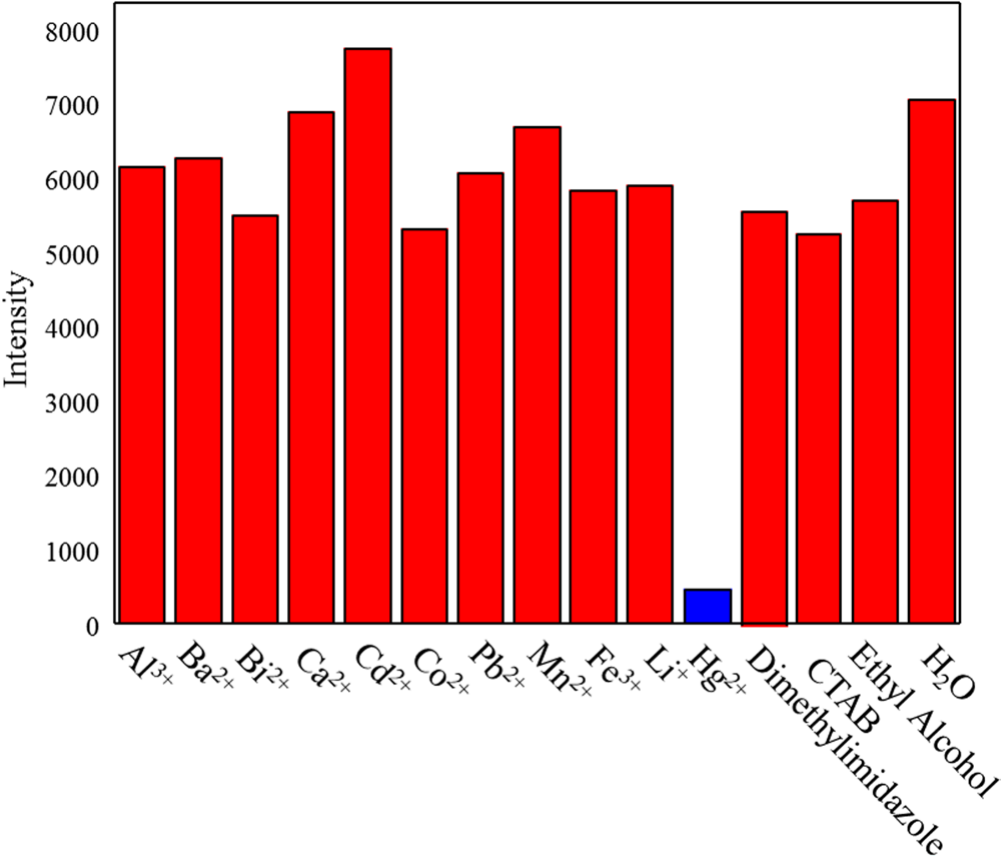
Fluorescence intensity of the FTP in response to a variety of solutes in aqueous solutions at a concentration of 0.1 M.

The FTP was tested to screen its response to a variety of solutes at a concentration of 0.1 M (Fig. 5). Among the tested solutes the fluorescence response to Hg^2+^ was quite remarkable, while quenching by other solutes did not occur, indicating a high selectivity of the FTP for Hg^2+^.

**Figure 6:**
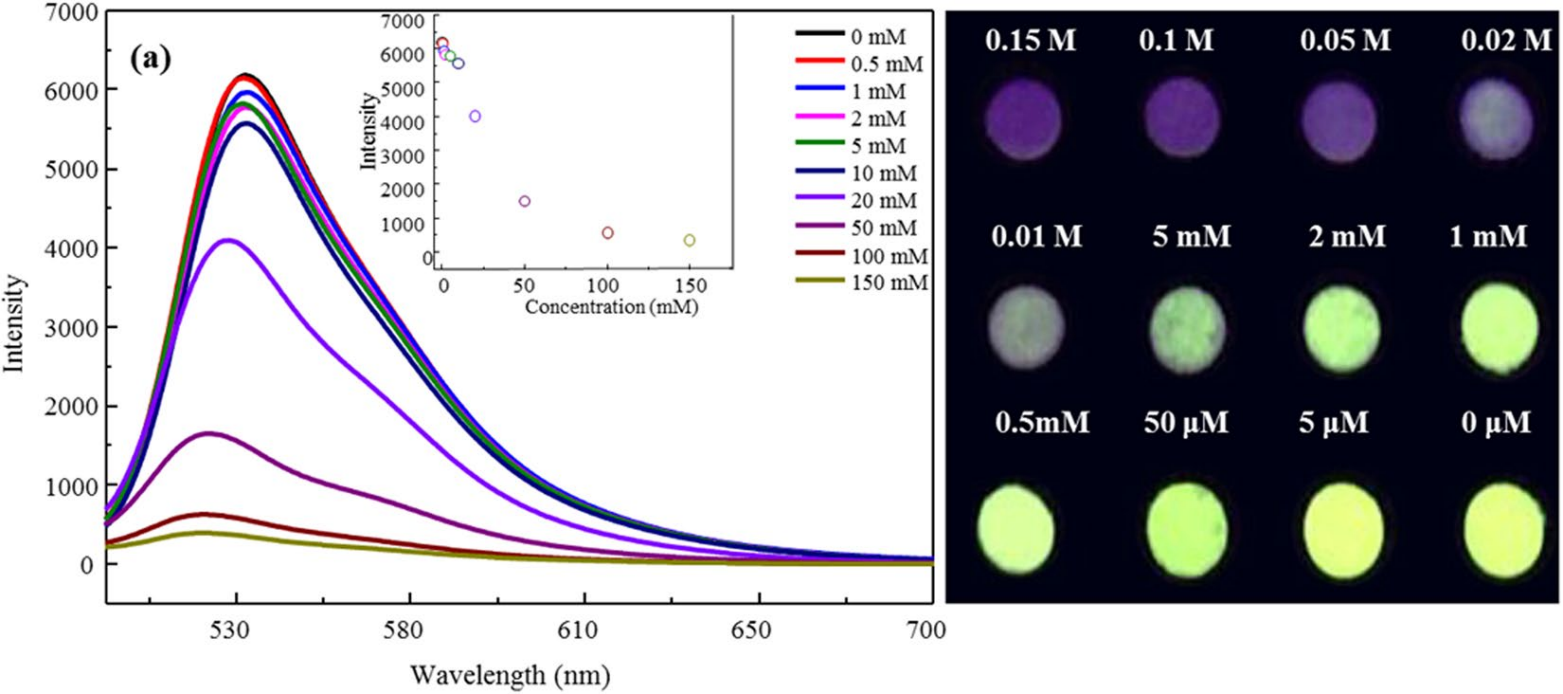
Fluorescence spectra of the FTP in response to different concentrations of Hg^2+^ (**a**). Images of the FTP with different concentrations of Hg^2+^ under ultraviolet light (**b**).

The photoluminescence (PL) quenching of FTP in the presence of Hg^2+^ in aqueous solution was further investigated as a function of initial Hg^2+^ concentration. Over the concentration range of 0.5–200 mM, an exponential decrease in fluorescence intensity was observed (Fig. 6a). A full PL quenching can be observed with naked eyes at an Hg^2+^ concentration of 50 mM. The detection limit of Hg^2+^ using FTP by naked eyes was 5 mM. The images of FTP with addition of Hg^2+^ under UV light showed a consistent tendency (Fig. 6b).

### Mechanism of fluorescence enhancement

**Figure 7:**
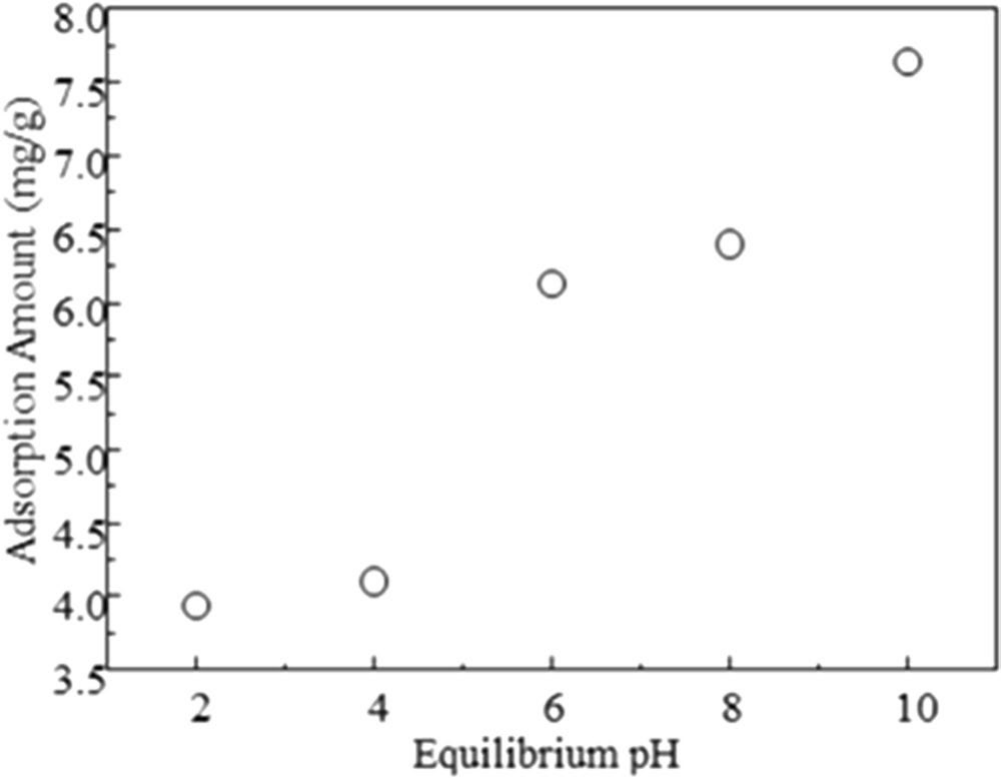
Influence of equilibrium solution pH on AO uptake on LDH.

Minute adsorption of AO from water by LDH was observed at an input concentration of 0.5–80 mg/L, resulting in an AO adsorption capacity of 6 mg/g that could be reached at an initial concentration of 40 mg/L. The LDH is an anion exchanger with positively charged surfaces while the AO is positively charged when the solution pH is lower than its pKa value of 10.4^24^. Thus, the influence of equilibrium solution pH on AO uptake was further assessed. As the pH increased from 2 to 10, the AO uptake increased progressively (Fig. 7). The total charge of a mineral is made of permanent charge, which is attributed to isomorphic substitution in crystal lattice (Mg^2+^ substitution by Al^3+^ in case of LDH^36^), and pH dependent charge, due to protonation/deprotonation^37^. The metal ions of LDH will dissolve under the condition of strong acidity (pH < 4) and its structure changes. LDH can be dissolved affecting its adsorption effect under the condition of strong acidity^38^. The phenomenon of LDH dissolution is not observed under other pH conditions. The adsorption of AO from pH 2 to 4 becomes better because of the less dissolution of LDH. The points of zero charge (PZC) refers the pH value at which the total charges was 0 under a certain temperature, pressure and medium. PZC can be further divided into point of zero net proton charge (PZNPC) and point of zero net charge (PZNC)^39^. The research found that hydrotalcite exists PZC^40^. The adsorption proton charge on the surface of hydrotalcite would change with the pH value of the medium. However, charges on the surface of LDH could change with the pH of system. The basal surfaces (perpendicular to crystallographic *c*) and prismatic surfaces (parallel to *c*) may show different affinity for AO molecules under different pH conditions due to electrostatic effect. The prismatic surface may bear negative charges under high pH due to deprotonation, which may result in an increased AO uptake (Fig. 7).

**Figure 8:**
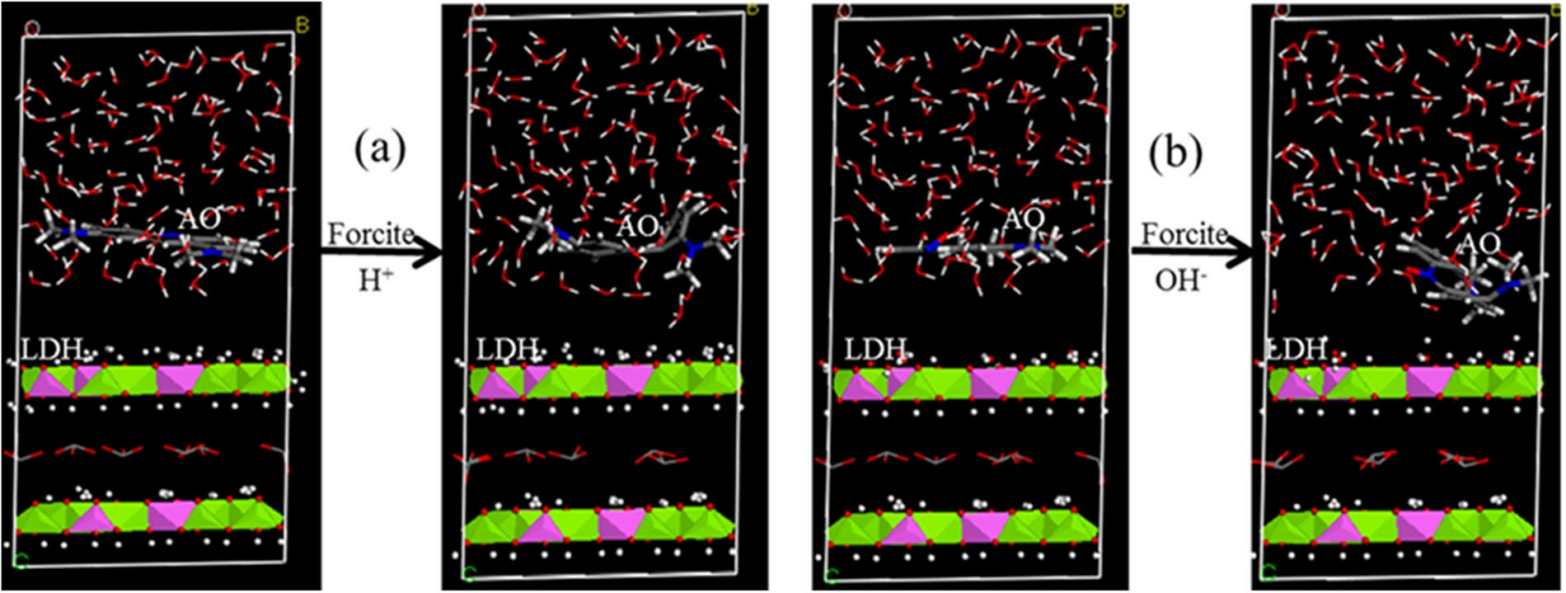
Molecular dynamic simulation of interactions between AO and the basal surfaces of LDH under acidic (**a**) and alkalinic (**b**) conditions. For all species, C = gray, N = blue, H = white, O = red, Mg = green, Al = pink.

**Figure 9:**
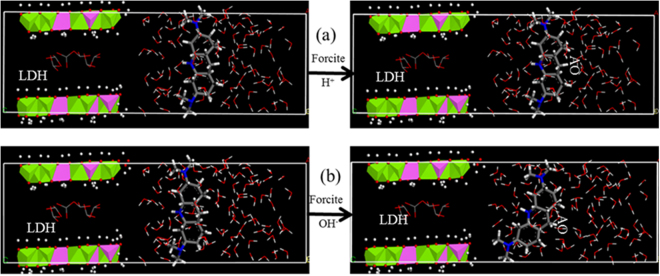
Molecular dynamic simulation of interactions between AO and the prismatic surfaces of LDH under acidic (**a**) and alkalinic (**b**) conditions. For all species, C = gray, N = blue, H = white, O = red, Mg = green, Al = pink.

To support the electrostatic interactions between AO and LDH surfaces, molecular dynamics simulation studies were performed under low and high pH conditions for the interactions between the basal or prismatic surfaces. The positive charge of AO is located at the dimethylammonium group. Under both low and high pH conditions the interactions between AO and the basal surface of LDH were all minimal (Fig. 8), suggesting that the basal surfaces were dominated by permanent surface charges, resulting in a net repulsion between the positively charged AO and the LDH basal surface. A similar result was found for the interactions between AO and prismatic surfaces of LDH under low pH condition (Fig. 9a). However, under a high pH condition, it seemed that the dimethylammonium group of AO moved towards the prismatic surfaces of LDH (Fig. 9b). Thus, it is very likely that the deprotonation of the prismatic surface of LDH played a crucial role in AO uptake.

## Conclusions

In this study, an organic dye acridine orange (AO) was successfully loaded on LDH surfaces. The maximal AO separation resulted in significantly inhibition in fluorescence quenching. With its enhanced fluorescent property, the composite powder was mixed with pulp to fabricate a fluorescence test paper, which can detect Hg^2+^ in water by naked eyes with a detection limit of 5 mM. Further optimization may find its practical application in environmental and wastewater treatment fields.

## Experimental Section

### Experimental materials

The Mg-Al-LDH was obtained from Ica Shanghai biological technology co., LTD (Russia) and was used as is without further purification. The AO (CAS # 65-61-2) used was in an HCl form purchased from Sigma-Aldrich. Its molecular formula is C_34_H_40_C_l4_N_6_Zn and its formula weight is 739.94. It had a pKa value of 10.4, below which the molecule would be protonated to form AOH^+^. NaOH, HCl, distilled water was brought from Beijing chemical factory. Mercuric chloride was from best reagent.

### Methods

For batch study, 0.1 g of LDH and 25 mL of AO solution at concentration of 0.5–80 mg/L was added to each 50 mL centrifuge tube. The mixtures were shaken at 300 rpm for 6 h and then were centrifuged at 7500 rpm for 2 min. The supernatants were then filtered through 0.22 μm syringe filters before being analyzed by a UV-vis spectrophotometer. The amount of AO adsorbed was determined from the differences between the initial and equilibrium concentrations. All experiments were done in duplicate. The solid product is denoted as AO/LDH. Similar experiments were done under different pH conditions.

TO fabricate the FTP, 1 g of pulp and varying amounts of AO/LDH at the pulp to AO/LDH ratios of 1:10, 1:5, 1:2, 1:1 and 2:3 were added together with 100 mL of distilled water to each 200 mL Erlenmeyer flask. The mixture was stirred for 10 h to increase the integration. The final product was named fluorescence test paper (FTP) with good flexibility, high luminescent property and controllability.

To assess the solute detection, the following solutions at a concentration of 0.1 M were initially screened for fluorescence quenching of AO: Al^3+^, Ba^2+^, Bi^2+^, Cd^2+^, Co^2+^, CTAB, Hg^2+^, Fe^3+^, Li^+^, dimethylimidazole, Mn^2+^, Pb^2+^, Ethyl Alcohol. They were dropped onto the FTP. Then, the fluorescence intensities were determined using a fluorescence spectrophotometer (HITACHI, F4600). To evaluate the Hg^2+^ detection response, drops of Hg^2+^ solutions of different concentrations were placed onto the FTP. The initial Hg^2+^ concentrations were from 0.5–200 mM. The florescence intensity was measured by a fluorescence spectrophotometer (HITACHI, F4600).

### Methods of analyses

The absorbance values of equilibrium solutions were measured using a UV-vis spectrophotometer (T6 New Century) at wavelength of 490 nm.

Powder XRD analyses were performed on a Rigaku D/max-IIIa diffractometer with a Ni-filtered CuKa radiation at 30 kV and 20 mA. Orientated samples were scanned from 3° to 70° at 8°/min with a scan step of 0.02°/step.

Scanning electron micrography (SEM) was recorded using a JEOL-IT300 Scanning electron microscope at an accelerating voltage of 10 kV.

Photoluminescence emission (PL) spectra were acquired on a fluorescence spectrophotometer (HITACHI, F4600) over the range of 500−700 nm with a photomultiplier tube operated at 800 V. A 150 W xenon lamp was used as the excitation source, at an excitation wavelength of 488 nm. The scan speed was set at 240 nm/min.

Molecular simulation was carried out under the module “Forcite” of Materials Studio 6.0 software to study the configuration of AO in the surface of LDH. The unit cell (R-3)^41^ parameters were set at a = b = 3.046 Å, c = 22.78 Å, a = 90°, γ = 90°, and b = 120°^42^. A series of 2 × 2 × 1 supercells were built. Three cycles were reiterated with each cycle made of 106 steps.
